# Multidimensional Healthcare Access Barriers to Prostate‐Specific Antigen Testing: A Nation‐Wide Panel Study in the United States From 2006 to 2020

**DOI:** 10.1002/cam4.70358

**Published:** 2024-11-06

**Authors:** Hari S. Iyer, Kevin H. Kensler, Charlotte Roscoe, Chidinma Opara, Mingchao He, Evan Kovac, Isla P. Garraway, Quoc Dien‐Trinh, Timothy R. Rebbeck

**Affiliations:** ^1^ Section of Cancer Epidemiology and Health Outcomes Rutgers Cancer Institute of New Jersey New Brunswick New Jersey USA; ^2^ Department of Population Health Sciences Weill Cornell Medical Center New York New York USA; ^3^ Division of Population Sciences Dana‐Farber Cancer Institute Boston Massachusetts USA; ^4^ Department of Environmental Health Harvard T. H. Chan School of Public Health Boston Massachusetts USA; ^5^ Rutgers Cancer Institute of New Jersey Newark New Jersey USA; ^6^ Department of Surgical and Perioperative Care Veterans Affairs Greater Los Angeles Healthcare System Los Angeles California USA; ^7^ Department of Urology David Geffen School of Medicine at University of California Los Angeles California USA; ^8^ Jonsson Comprehensive Cancer Center at University of California Los Angeles California USA; ^9^ Center for Surgery and Public Health Brigham & Women's Hospital Boston Massachusetts USA; ^10^ Department of Epidemiology Harvard T. H. Chan School of Public Health Boston Massachusetts USA

**Keywords:** accessibility of health services, cancer screening, prostate‐specific antigen, prostatic neoplasms, racial groups

## Abstract

**Background:**

Rising metastatic prostate cancer incidence has renewed debate regarding benefits of prostate‐specific antigen (PSA) screening. Identifying barriers to accessing screening for individuals at high risk of lethal prostate cancer may slow this rise. We examined associations of access barriers with receipt of PSA testing, stratified by sociodemographic factors.

**Methods:**

We pooled data from male respondents to Behavior Risk Factor Surveillance Systems (BRFSS) surveys from 2006 to 2020. Questions related to affordability (insurance, cost of visits) and accommodation (regular primary care provider (PCP), physician recommending a PSA test) were considered as individual‐level barriers. For availability, we linked provider density from the 2012 Area Health Resource File and estimated driving times to closest health facility within Micropolitan and Metropolitan Statistical Area (MMSA) using Google Earth Engine. These measures were used to compute a spatial accessibility index. We fit survey‐weighted, covariate‐adjusted logistic regression models to estimate associations of barriers with receipt of PSA within the past 2 years and examined effect modification by sociodemographic factors.

**Results:**

There were 185,643 participants, of whom 73% were White, 11% were Black, 4% were Asian, and 11% were Hispanic. Physician recommendation was the strongest predictor of having a PSA test (aOR: 14.5, 95% CI: 13.6, 15.6). Not having a regular PCP (aOR: 0.29, 95% CI: 0.27, 0.31), insurance (aOR: 0.64, 95% CI: 0.58, 0.71), and prohibitive cost of care (aOR: 0.82, 95% CI: 0.75, 0.90) were associated with lower PSA testing. Access barriers were stronger predictors of PSA testing for Asian and White participants compared to other groups (*P*_het_ < 0.004 for insurance and regular PCP) and for those with college education compared to those without (*P*_het_ < 0.05 for insurance, perceived unaffordability).

**Discussion:**

Physician recommendation was the strongest predictor of receipt of PSA testing, regardless of sociodemographic grouping. Future studies should consider access barriers jointly and across sociodemographic strata.

## Abstract 2

Understanding barriers to accessing appropriate prostate‐specific antigen screening is important in light of concerning increases in metastatic prostate cancer incidence. While insurance, regular contact with a primary care provider, and high cost were barriers to accessing screening in a panel study using Behavior Risk Factor Surveillance System data, physician's recommendation was the strongest predictor of screening.

## 1 Introduction

Prostate cancer (CaP) is a leading cause of cancer‐related morbidity and mortality in US men, accounting for an estimated 288,300 new cases and 34,700 deaths per year [1]. Age, genetics, and Black race are the strongest predictors of CaP [2, 3]. Higher incidence of CaP in Black individuals, particularly for aggressive and fatal CaP, may arise from joint influences of genetic susceptibility and environmental factors including stressors arising from discrimination and socioeconomic disadvantage and lower access to high‐quality healthcare [4, 5, 6, 7, 8, 9, 10]. Following decades of decline, metastatic CaP incidence has begun to rise [11]. The lack of strong, reliable modifiable risk factors for CaP makes targeted early detection and linkage to appropriate care one of the only effective means for halting this concerning trend [2, 12].

The most widely used screening modality for CaP is prostate‐specific antigen (PSA) testing. PSA screening for CaP is controversial due to mixed evidence from randomized clinical trials regarding benefits [13, 14, 15, 16]. While the European Randomized study of Screening for Prostate Cancer (ERSPC) reported a 20% relative reduction in mortality from screening over 16 years of follow‐up [17] evidence from the largest American study, the Prostate, Lung, Colorectal, and Ovarian (PLCO) cancer screening trial showed no benefits, in part due to high levels of screening in the control (usual care) arm [13, 18]. Based on these findings, the US Preventive Services Task Force (USPSTF) advocated against screening from 2012 through 2018 [15]. In 2018, following early evidence showing increases in the relative burden of late‐stage CaP and CaP‐specific mortality [11, 19], the USPSTF shifted their position by advocating shared decision‐making for CaP screening [20].

Most CaP experts now agree that the harm–benefit trade‐offs of PSA screening can be optimized by targeting men at high risk for aggressive disease based on genetics, family history, Black race, and age [21, 22]. Notably, men with these risk factors remain understudied in clinical trials, with only 4% of the PLCO cohort self‐identifying as Black or African American [23] and no ethnicity data provided in the ESRPC [17]. However, members within those high‐risk groups may continue to experience barriers that prevent them from accessing appropriate screening [24, 25, 26, 27]. Although Black men represent a known high‐risk group that may disproportionately experience access barriers associated with low socioeconomic status and racial discrimination, members of other racial and ethnic groups may have higher risk of aggressive CaP due to genetics or obesity [5, 28, 29]. While prior studies have investigated associations of sociodemographic groups with receipt of PSA testing [30, 31], fewer studies have considered whether the importance of specific access barriers may vary based on sociodemographic characteristics [32, 33]. Understanding which access barriers are most important for different racial and ethnic or socioeconomic groups of patients could improve identification of high‐risk men within those groups, leading to higher uptake of appropriate CaP screening [34]. Identifying barriers could also support future trial recruitment strategies in men at greater susceptibility to prostate cancer.

In order to address these gaps, we studied associations of multidimensional barriers to accessing screening for CaP in a nation‐wide, repeated survey. We then sought to examine whether associations varied by sociodemographic characteristics.

## 2 Materials and Methods

### 2.1 Study Population and Design

Participant data were obtained from the Behavior Risk Factor Surveillance System (BRFSS). The BRFSS is a state‐level, nation‐wide annual survey conducted by state health departments with technical and methodological support from the United States Center for Disease Control and Prevention [35, 36]. This survey generates data for monitoring state and national trends in healthcare behaviors, including screening for cancer. The BRFSS is conducted through telephone interviews using Random Digit Dialing sampling. Although early surveys were conducted using landline phones, in recent times, cellphones have been added to the sampling frame. Due to changes in population‐based sampling weights, later prevalence estimates are not comparable with data collected prior to 2011. For this reason, in our study, we have chosen not to present time‐varying trends in screening.

Questions about cancer screening are asked every 2 years in the BRFSS. For this study, we included data from the 2006, 2008, 2010, 2012, 2014, 2016, 2018, and 2020 surveys, which included questions about screening for CaP. Data from 2022 were not included due to potential unmeasured influences of COVID‐19 lockdowns on preventive healthcare seeking. Of 1,910,304 total respondents, we excluded female respondents (*n* = 1,199,736), those reporting a prior history of CaP (*n* = 19,736), with missing responses to questions on access barriers and marital status (*n* = 164), receipt of PSA testing (*n* = 58,889), and those aged < 55 or > 69 years to match ages currently recommended for PSA screening [37]. We further excluded those self‐identifying as Indigenous American or Pacific Islander race (*n* = 23,625) due to potential selection bias due to non‐response [38] and statistical imprecision of odds ratio estimates, resulting in 185,643 participants for analysis. Starting in 2006, geographic identifiers for Metro‐ and Micropolitan Statistical Areas (MMSAs) were provided for respondents residing in 1893 regions of the United States as part of the Selected Metropolitan/Micropolitan Area Risk Trends (SMART) database. We used MMSA geography to link socioeconomic and health services data to BRFSS participants. This study was deemed exempt from IRB review as a secondary analysis of deidentified data.

### 2.2 Measures of Access Barriers

Penchansky and Thomas proposed five dimensions of access: physical access, availability, affordability, accommodation, and acceptability [24]. Geographic data can be used to develop measures of physical access and availability, and so these barriers are often jointly referred to as spatial accessibility [39]. Affordability refers to costs of services and ability of patients to pay. Accommodation refers to the convenience of appointments for the patient. Acceptability refers to whether the patient feels comfortable and treated with respect. Together, all of these factors play a role in ensuring that patients can receive the care they need [24].

We organized responses to questions about healthcare access in the BRFSS by access dimension. We included the questions “Was there a time in the past 12 months when you needed to see a doctor but could not because of cost?” and “Do you have any kind of health care coverage, including health insurance, prepaid plans such as HMOs, or government plans such as Medicare?” as capturing affordability barriers. We included the questions “Do you have one person you think of as your personal doctor or healthcare provider?” as proxy measures to capture accommodation barriers. We coded variables to indicate presence of a barrier (e.g., having no healthcare coverage, not seeing a doctor, and not having a personal doctor). In addition, from 2012 to 2020 during even years, BRFSS surveys asked male respondents additional questions about their use of PSA testing for CaP screening. We additionally included data from responses to the question “Has a doctor ever recommended that you have a PSA test?” as a proxy measure for accommodation.

For spatial accessibility, we obtained data from the 2012 Area Health Resource File [40], a government database capturing detailed information on health systems inputs (staff, patient visits, and hospital equipment) and sociodemographic data at MMSA level. We estimated average travel time to the nearest facility within each MMSA using a friction‐based least cost distance algorithm implemented by Weiss et al. within Google Earth Engine to obtain estimates of travel times to healthcare facilities worldwide [41]. This method does not calculate travel time between an origin and destination, but instead computes a grid with cells 1 km × 1 km, where the time assigned to each cell corresponds to the minimum time required for a person living in that cell to reach their closest facility. In brief, this method leveraged data from the Open Street Map database of healthcare facilities classified as hospitals and clinics (*n* = 148,522) [42], data on facility locations extracted from Google Maps (*n* = 201,799) [43], and national facility assessments [44, 45] in 2019. The “friction surface” was calculated using geographic databases of land cover, elevation, topographic conditions, and roads, which assigned a “cost” to traveling across each surface type. The least cost distance method was implemented in Google Earth Engine using the cumulativeCost() function, which tests all routes from every 1 km × 1 km cell to every healthcare facility, and assigns the minimum time to reach the closest facility to that cell. Validation was performed by comparing routes estimated from the least cost distance method to trips from origins and destinations entered into Google Maps' API [46]. Further details about the method are provided elsewhere [41].

We calculated a MMSA‐level PSA spatial accessibility index. We calculated the density of urologists, PCPs, and radiation oncologists per 1000 in each MMSA as proxies for availability of CaP screening and referral services. Although urologists and radiation oncologists do not directly offer screening, they represent an important outcome of screening (prompt referral for treatment). We first estimated the average travel times across grid cells within each MMSA using Google Earth Engine. We then calculated quartiles for each input, and summed to generate the index, which ranged from 0 (lowest spatial accessibility) to 16 (highest spatial accessibility). We flipped the direction of quartiles for the travel time measure so interpretation was consistent for all components.

### 2.3 Prostate‐Specific Antigen Testing

Our goal was to model correlates of receipt of PSA tests for screening. Unfortunately, the indication for the test was not captured in all of the BRFSS surveys used for this analysis. We therefore used responses to the questions “Have you ever had a PSA test?” and “How long has it been since you had your last PSA test?” We created a binary variable for receipt of PSA test within 2 years, and assumed that tests among those not diagnosed with CaP were screening tests, following convention [31].

### 2.4 Statistical Analysis

We applied survey weights to estimate population‐based frequencies and measures of effect and to account for potential sampling biases in our effect estimates. We then summarized sociodemographic, behavioral, and healthcare access barrier measures across racial and ethnic categories and receipt of PSA testing using two‐sample independent t‐tests, non‐parametric Wilcoxon rank sum tests, and chi‐squared tests for independence. In order to estimate associations of access barriers on receipt of PSA testing, we fit a series of logistic regression models to estimate odds ratios for associations with each access barrier measure and receipt of PSA testing: (Model 1) unadjusted; (Model 2) multivariable logistic regression models, adjusting for age [5‐year categories: 40–44, 45–49, 50–54, 55–59, 60–64, 65–69, 70–74, 75–79, 80, or older], race and ethnicity [categorical: non‐Hispanic White (NHW), non‐Hispanic Black (NHB), Asian, and Hispanic], survey year [categorical], education [categorical: less than high school, high school/some college, college or higher], income [categorical, $ per year: < $35,000, $35,000–$74,999, ≥ $75,000], marital status [categorical: married/cohabiting, divorced/widowed/separated, never married], current smoking [categorical: yes, no], binge drinking [categorical: yes, no], reported days of poor physical health [categorical: none, 1–13, 14–30], reported days of poor mental health [none, 1–13, 14–30]. For analyses of spatial accessibility, we further adjusted for a MMSA‐level index of neighborhood socioeconomic status using quintiles, calculated from a previously described z‐score‐based index of component census‐derived measures of income, education, wealth, occupation, and demographic factors assessed from the 2006 to 2010 American Community Survey [47, 48]. We repeated analyses by exposure in unadjusted and fully adjusted models, further adjusting for individual‐level access barriers (insurance, cost, routine checkup, and regular care provider). For covariates, we treated responses of “do not know,” “not sure,” “refused,” and “missing” as missing using a missing indicator.

In order to evaluate effect modification, we conducted analyses stratified by race and ethnicity, educational attainment, and the MMSA‐level neighborhood socioeconomic status index (quintiles). We fit multiplicative interaction terms between each modifier and each barrier and used likelihood ratio tests for heterogeneity to evaluate statistical significance. All significance tests were two‐sided with Type‐1 error rate of 0.05. Analyses were run using R version 4.2.1.

## 3 Results

### 3.1 Participant Characteristics

Our sample included 185,643 men, which corresponded to a population‐weighted estimate of 94,023,926 male individuals across surveys. Most (38%) of the population was aged 55–59, and 73% were NHW, 11% were NHB, 4% were Asian, and 11% were Hispanic (Table 1). Most (73%) were married or cohabiting, 39% had college or higher education, with 64% having incomes of $35,000 per year or more. Most participants were non‐smokers (83%) and non‐binge drinkers (85%).

**TABLE 1:** Characteristics of male respondents to the Behavior Risk Factor Surveillance System, 2006–2020 (weighted population).

	Non‐Hispanic White	Non‐Hispanic Black	Asian	Hispanic	Overall
*N* (%)	68,396,822.9 (73%)	10,775,310.9 (11%)	4,114,750 (4%)	10,737,042 (11%)	94,023,925.8 (100%)
Age group (years) (%)					
55–59	25,002,546.6 (36.6)	4,225,295.5 (39.2)	1,494,902.4 (36.3)	4,549,722.3 (42.4)	35,272,466.9 (37.5)
60–64	24,207,252.7 (35.4)	3,644,165.2 (33.8)	1,316,433.3 (32.0)	3,470,033.3 (32.3)	32,637,884.4 (34.7)
65–69	17,644,260.5 (25.8)	2,559,063.5 (23.7)	1,053,063.2 (25.6)	2,468,642.4 (23.0)	23,725,029.8 (25.2)
Do not know/not sure or missing	1,542,763.1 (2.3)	346,786.7 (3.2)	250,351.0 (6.1)	248,644.0 (2.3)	2,388,544.7 (2.5)
Education (%)					
Less than high school	3,632,389.8 (5.3)	1,820,116.1 (16.9)	250,402.9 (6.1)	4,318,867.5 (40.2)	10,021,776.3 (10.7)
High school/some college	35,342,513.9 (51.7)	6,517,810.7 (60.5)	1,314,840.5 (32.0)	4,336,198.5 (40.4)	47,511,363.6 (50.5)
College or higher	29,273,327.3 (42.8)	2,372,520.6 (22.0)	2531869.2 (61.5)	2051011.1 (19.1)	36,228,728.2 (38.5)
Missing	14,8591.9 (0.2)	64,863.5 (0.6)	17,637.4 (0.4)	309,64.9 (0.3)	262,057.7 (0.3)
Income (%)					
< $35,000	13,253,446.6 (19.4)	4,475,981.9 (41.5)	987,146.1 (24.0)	5,894,673.4 (54.9)	24,611,248.0 (26.2)
$35,000–$75,499	19,035,003.9 (27.8)	2,698,096.3 (25.0)	848,843.7 (20.6)	2,168,338.2 (20.2)	24,750,282.1 (26.3)
≥ $75,000	29,262,661.7 (42.8)	2,375,228.2 (22.0)	1,765,933.1 (42.9)	1,622,214.9 (15.1)	35,026,037.9 (37.3)
Missing	6,845,710.6 (10.0)	1,226,004.5 (11.4)	512,827.1 (12.5)	1,051,815.5 (9.8)	9,636,357.7 (10.2)
Census region (%)					
Northeast	14,578,582.8 (21.3)	2,111,885.5 (19.6)	950,289.7 (23.1)	1,733,790.9 (16.1)	19,374,548.9 (20.6)
Midwest	16,339,368.2 (23.9)	2,153,550.7 (20.0)	320,406.5 (7.8)	924,878.0 (8.6)	19,738,203.4 (21.0)
South	23825187.7 (34.8)	5496787.7 (51.0)	856237.6 (20.8)	3591941.8 (33.5)	33,770,154.9 (35.9)
West	13,651,142.7 (20.0)	1,011,246.7 (9.4)	1,987,816.1 (48.3)	3,759,956.9 (35.0)	20,410,162.5 (21.7)
Missing	2541.4 (0.0)	1840.3 (0.0)	0.0 (0.0)	726,474.3 (6.8)	730,856.0 (0.8)
Marriage (%)					
Married/cohabiting	51,330,123.7 (75.0)	5,943,222.9 (55.2)	3,472,196.5 (84.4)	7,701,385.9 (71.7)	68,446,929.1 (72.8)
Divorced/widowed/separated	11,667,900.0 (17.1)	3,193,163.0 (29.6)	373,693.4 (9.1)	2,275,041.2 (21.2)	17,509,797.6 (18.6)
Never married	5,152,045.5 (7.5)	1,578,954.4 (14.7)	245,549.0 (6.0)	726,837.1 (6.8)	7,703,386.0 (8.2)
Refused	246,753.7 (0.4)	59,970.7 (0.6)	23,311.0 (0.6)	33,777.8 (0.3)	363,813.1 (0.4)
Employment (%)					
Employed	39,150,329.6 (57.2)	4,596,222.5 (42.7)	2,550,972.3 (62.0)	5,637,772.4 (52.5)	51,935,296.8 (55.2)
Not employed	8,277,648.2 (12.1)	2,657,159.3 (24.7)	526,952.2 (12.8)	2,386,591.9 (22.2)	13,848,351.6 (14.7)
Retired	20,748,593.0 (30.3)	3,457,960.1 (32.1)	984,001.9 (23.9)	2,642,322.5 (24.6)	27,832,877.6 (29.6)
Refused	220,252.0 (0.3)	63,969.0 (0.6)	52,823.6 (1.3)	70,355.2 (0.7)	407,399.8 (0.4)
Current smoking (%)					
Yes	10,592,081.5 (15.5)	2,734,523.6 (25.4)	4,25,536.7 (10.3)	1,526,885.4 (14.2)	15,279,027.2 (16.3)
No	57,511,748.1 (84.1)	7,982,315.2 (74.1)	3,651,539.7 (88.7)	9,091,066.5 (84.7)	78,236,669.5 (83.2)
Do not know/refused/missing	292,993.3 (0.4)	58,472.2 (0.5)	37,673.5 (0.9)	119,090.1 (1.1)	508,229.1 (0.5)
Binge drinking (%)					
Yes	9,104,648.3 (13.3)	1,175,352.5 (10.9)	269,751.5 (6.6)	1,540,876.4 (14.4)	12,090,628.7 (12.9)
No	58,073,816.6 (84.9)	9,284,206.1 (86.2)	3,787,148.3 (92.0)	8,922,019.0 (83.1)	80,067,189.9 (85.2)
Do not know/refused/missing	1,218,358.0 (1.8)	315,752.3 (2.9)	57,850.2 (1.4)	274,146.7 (2.6)	1,866,107.1 (2.0)
Days with poor physical health (%)					
None	45,020,542.1 (65.8)	6,274,552.4 (58.2)	2,867,880.9 (69.7)	6,368,592.4 (59.3)	60,531,567.9 (64.4)
1–13	13,668,535.7 (20.0)	2,220,934.0 (20.6)	777,561.0 (18.9)	2,071,092.8 (19.3)	18,738,123.4 (19.9)
14–30	8,772,873.2 (12.8)	2,008,501.8 (18.6)	354,655.9 (8.6)	1,976,513.1 (18.4)	13,112,543.9 (13.9)
Missing	934,871.9 (1.4)	271,322.7 (2.5)	114,652.2 (2.8)	320,843.8 (3.0)	1,641,690.6 (1.7)
Days with poor mental health (%)					
None	52,295,706.2 (76.5)	7,728,293.3 (71.7)	3,313,646.7 (80.5)	7,745,503.2 (72.1)	71,083,149.3 (75.6)
1–13	10,012,542.9 (14.6)	1,633,452.6 (15.2)	529,269.4 (12.9)	1,589,920.4 (14.8)	13,765,185.3 (14.6)
14–30	5,323,920.7 (7.8)	1,208,599.5 (11.2)	216,987.1 (5.3)	1,152,002.2 (10.7)	7,901,509.5 (8.4)
Missing	764,653.2 (1.1)	204,965.5 (1.9)	54,846.7 (1.3)	249,616.2 (2.3)	1,274,081.6 (1.4)
Has insurance coverage (%)					
Yes	64,328,498.9 (94.1)	9,436,628.6 (87.6)	3,736,719.4 (90.8)	8,501,555.6 (79.2)	86,003,402.5 (91.5)
No	3,944,654.5 (5.8)	1,309,134.2 (12.1)	356,826.0 (8.7)	2,195,373.8 (20.4)	7,805,988.5 (8.3)
Do not know/not sure	57,374.9 (0.1)	14,961.4 (0.1)	21,204.6 (0.5)	11,598.6 (0.1)	105,139.4 (0.1)
Refused	66,294.6 (0.1)	14,586.7 (0.1)	0.0 (0.0)	28,514.0 (0.3)	109,395.3 (0.1)
Regular primary care provider (%)					
Yes, only one	5,667,9810.2 (82.9)	8,462,615.7 (78.5)	3,341,584.1 (81.2)	7,509,347.3 (69.9)	75,993,357.3 (80.8)
More than one	4,940,403.0 (7.2)	890,588.0 (8.3)	260,344.1 (6.3)	745,536.5 (6.9)	6,836,871.6 (7.3)
No	6,634,307.8 (9.7)	1,362,452.8 (12.6)	492,951.2 (12.0)	2,432,884.1 (22.7)	10,922,595.9 (11.6)
Do not know/not sure	87,298.3 (0.1)	44,402.5 (0.4)	18,892.5 (0.5)	28,022.6 (0.3)	178,615.9 (0.2)
Refused	55,003.7 (0.1)	15,251.9 (0.1)	978.0 (0.0)	21,251.5 (0.2)	92,485.1 (0.1)
Could not see doctor due to cost (%)					
Yes	4,544,216.3 (6.6)	1,378,078.6 (12.8)	484,362.0 (11.8)	1,812,111.6 (16.9)	8,218,768.5 (8.7)
No	63,749,475.9 (93.2)	9,352,679.7 (86.8)	3,600,253.8 (87.5)	8,890,709.1 (82.8)	85,593,118.5 (91.0)
Do not know/not sure	72,179.7 (0.1)	28,948.0 (0.3)	24,589.9 (0.6)	27,621.3 (0.3)	153,338.9 (0.2)
Refused	30,951.0 (0.0)	15,604.7 (0.1)	5544.3 (0.1)	6599.9 (0.1)	58,699.9 (0.1)
Duration of time since last check up (%)					
Within past year	52,976,600.4 (77.5)	9,110,014.8 (84.5)	3,270,251.5 (79.5)	7,792,470.8 (72.6)	73,149,337.6 (77.8)
Within past 2 years	7,097,864.1 (10.4)	811,046.5 (7.5)	442,869.9 (10.8)	1,223,416.8 (11.4)	9,575,197.3 (10.2)
Within past 5 years	3,782,253.1 (5.5)	364,582.4 (3.4)	185,813.0 (4.5)	783,065.3 (7.3)	5,115,713.8 (5.4)
5 or more years ago	3,804,744.1 (5.6)	354,575.6 (3.3)	150,082.1 (3.6)	618,336.3 (5.8)	4,927,738.1 (5.2)
Do not know/not sure	346,646.0 (0.5)	59,976.5 (0.6)	26,364.0 (0.6)	71,758.2 (0.7)	504,744.7 (0.5)
Never	337,823.9 (0.5)	66,943.6 (0.6)	31,109.7 (0.8)	233,740.0 (2.2)	669,617.1 (0.7)
Refused	50891.3 (0.1)	8171.5 (0.1)	8259.8 (0.2)	14254.7 (0.1)	81,577.3 (0.1)
nSES (%)					
Q1	9,007,925.3 (13.2)	1,074,631.9 (10.0)	90,481.1 (2.2)	1,769,002.8 (16.5)	11,942,041.1 (12.7)
Q2	15,551,861.0 (22.7)	2,245,429.5 (20.8)	354,067.1 (8.6)	1,302,192.3 (12.1)	19,453,550.0 (20.7)
Q3	14,489,391.1 (21.2)	2,204,938.3 (20.5)	321,925.0 (7.8)	1,198,518.7 (11.2)	18,214,773.1 (19.4)
Q4	12,959,787.8 (18.9)	1,887,264.4 (17.5)	726,718.4 (17.7)	1,571,297.8 (14.6)	17,145,068.4 (18.2)
Q5	16,387,857.8 (24.0)	3,363,046.8 (31.2)	2,621,558.3 (63.7)	4,896,030.4 (45.6)	27,268,493.2 (29.0)
Race ICE (%)					
Q1	17,048,227.1 (24.9)	4,383,007.2 (40.7)	2,205,157.0 (53.6)	5,962,468.4 (55.5)	29,598,859.7 (31.5)
Q2	17,436,991.1 (25.5)	3,239,352.4 (30.1)	1,194,188.0 (29.0)	3,008,506.5 (28.0)	24,879,038.0 (26.5)
Q3	14,250,806.3 (20.8)	1,905,752.5 (17.7)	495,783.4 (12.0)	1,227,153.7 (11.4)	17,879,495.9 (19.0)
Q4	11,312,154.6 (16.5)	811,581.6 (7.5)	163,327.6 (4.0)	390,253.2 (3.6)	12,677,317.1 (13.5)
Q5	8,348,643.7 (12.2)	435,617.2 (4.0)	56,293.8 (1.4)	148,660.3 (1.4)	8,989,215.1 (9.6)
Income ICE (%)					
Q1	11,103,532.4 (16.2)	1,498,368.5 (13.9)	88,310.0 (2.1)	1,616,093.9 (15.1)	14,306,304.8 (15.2)
Q2	14,038,803.8 (20.5)	248,9421.8 (23.1)	301,313.1 (7.3)	1,462,082.3 (13.6)	18,291,621.0 (19.5)
Q3	17018504.4 (24.9)	2271616.3 (21.1)	586416.8 (14.3)	2539890.5 (23.7)	22,416,428.0 (23.8)
Q4	14,051,807.9 (20.5)	1,892,196.4 (17.6)	1,409,564.4 (34.3)	3,017,716.0 (28.1)	20,371,284.7 (21.7)
Q5	12,184,174.4 (17.8)	2,623,708.0 (24.3)	1,729,145.6 (42.0)	2,101,259.2 (19.6)	18,638,287.2 (19.8)
Racialized income ICE (%)					
Q1	11,521,328.5 (16.8)	2,837,625.2 (26.3)	268,155.2 (6.5)	1,597,902.0 (14.9)	16,225,010.9 (17.3)
Q2	16,835,575.9 (24.6)	2,004,601.3 (18.6)	563,616.2 (13.7)	2,986,607.9 (27.8)	22,390,401.4 (23.8)
Q3	17,495,068.6 (25.6)	2,398,890.1 (22.3)	1,199,056.6 (29.1)	3,428,312.2 (31.9)	24,521,327.6 (26.1)
Q4	13,446,803.0 (19.7)	2,427,129.1 (22.5)	1,308,045.3 (31.8)	1,930,675.2 (18.0)	19,112,652.6 (20.3)
Q5	9,098,046.8 (13.3)	1,107,065.1 (10.3)	775,876.7 (18.9)	793,544.6 (7.4)	11,774,533.2 (12.5)
Spatial accessibility index (%)					
Q1	20,042,292.8 (29.3)	3,485,514.9 (32.3)	849,639.4 (20.6)	3,229,242.3 (30.1)	27,606,689.4 (29.4)
Q2	14,346,333.3 (21.0)	1,825,073.7 (16.9)	864,535.1 (21.0)	2,319,573.5 (21.6)	19,355,515.7 (20.6)
Q3	21,209,158.4 (31.0)	3,336,889.0 (31.0)	1,592,940.1 (38.7)	3,591,194.5 (33.4)	29,730,182.0 (31.6)
Q4	12,799,038.4 (18.7)	2127833.3 (19.7)	807635.3 (19.6)	1597031.7 (14.9)	17331538.7 (18.4)
Urologist density per 1000 (median [IQR])	0.04 [0.03, 0.04]	0.04 [0.03, 0.05]	0.03 [0.03, 0.04]	0.03 [0.03, 0.04]	0.04 [0.03, 0.04]
FQHC density per 1000 (median [IQR])	0.01 [0.01, 0.02]	0.01 [0.01, 0.01]	0.01 [0.01, 0.01]	0.01 [0.00, 0.01]	0.01 [0.01, 0.01]
Outpatient visits per 1000 (median [IQR])	2110.12 [1379.61, 2673.28]	1975.30 [1326.33, 2488.12]	1495.56 [1277.48, 2285.38]	1415.15 [1267.62, 2230.09]	2043.50 [1292.00, 2612.07]
Average travel time to closest facility (minutes, median [IQR])	10.03 [7.86, 15.53]	8.94 [6.98, 13.21]	10.31 [6.98, 13.67]	12.12 [7.86, 21.29]	10.03 [7.40, 15.53]

Individual‐level socioeconomic status and health behaviors varied by race and ethnicity (Table 1). Compared to NHB (22%) and Hispanic (15%) respondents, NHW (43%) and Asian (43%) respondents were more likely to report income ≥ $75,000. NHB (19%) and Hispanic (18%) respondents were more likely than NHW (13%) and Asian (9%) respondents to report 14–30 days with poor physical health.

The estimate of overall screening prevalence from 2006 to 2020 was 58%, which declined to 51% in the subset who responded from 2012 to 2020. In analyses restricted to the participants surveyed in 2012–2020 (*n* = 115,292, weighted *n* = 58,553,203), most men had a PSA test as part of a routine examination, but this varied by race. Hispanic (43%) and Asian (42%) respondents were less likely than NHW (53%) and NHB (48%) respondents to report a doctor recommending a PSA test.

### 3.2 Multivariable Associations of Access Barriers With Receipt of PSA Test

Odds ratios for individual barriers attenuated following adjustment for sociodemographic and behavioral covariates, and upon adjustment for other barriers (Table 2). Having no insurance was associated with 35% lower odds of receiving a PSA test within 2 years (aOR: 0.64, 95% CI: 0.58, 0.71). Participants who reported that high cost prevented them from seeing a care provider had lower odds of receiving PSA tests (aOR: 0.82, 95% CI: 0.75, 0.90). Participants who did not have a regular PCP had lower odds of receiving screening (aOR: 0.29, 95% CI: 0.27, 0.31). Higher MMSA‐level spatial accessibility index was associated with lower receipt of PSA testing in the total population (Quintile 4 vs. 1: aOR: 0.86, 95% CI: 0.81, 0.91). Patterns were similar among the subset surveyed between 2012 and 2020 (Table S1). In the subgroup that was asked additional questions about PSA screening, there was a 14‐fold increase (aOR: 14.5, 95% CI: 13.6, 15.6) in the odds of receiving a PSA test in the prior 2 years associated with a doctor's recommendation.

**TABLE 2:** Associations of individual‐level barriers to accessing care and receipt of PSA test within 2 years in single and joint exposure models (2006–2020).

	Single		Multiple	
	Unadjusted	Adjusted^a^	Unadjusted^b^	Adjusted^a^
Barrier	OR (95% CI)	aOR (95%C CI)	OR (95% CI)	aOR (95%C CI)
No insurance	0.30 (0.27, 0.32)	0.44 (0.40, 0.49)	0.51 (0.46, 0.56)	0.64 (0.58, 0.71)
Unaffordable	0.44 (0.40, 0.47)	0.66 (0.60, 0.72)	0.61 (0.56, 0.66)	0.82 (0.75, 0.90)
No regular PCP	0.21 (0.19, 0.22)	0.26 (0.24, 0.28)	0.25 (0.24, 0.27)	0.29 (0.27, 0.31)
Doctor recommended PSA test^c^	17.82 (16.67, 19.06)	15.12 (14.12, 16.2)	16.44 (15.36, 17.6)	14.55 (13.57, 15.6)
Spatial access index				
Q1 (low access)	Ref	Ref	Ref	Ref
Q2	0.92 (0.87, 0.97)	0.95 (0.89, 1.01)	0.92 (0.87, 0.98)	0.96 (0.90, 1.02)
Q3	0.92 (0.88, 0.96)	1.02 (0.97, 1.07)	0.89 (0.85, 0.94)	1.01 (0.96, 1.07)
Q4 (high access)	0.92 (0.87, 0.97)	0.89 (0.84, 0.94)	0.87 (0.83, 0.92)	0.86 (0.81, 0.91)

^a^ Logistic regression models adjusted for age, race, survey year, education, income, marital status, employment, smoking, binge drinking, reported days of poor physical health, and reported days of poor mental health, census divisions.

^b^ Other barriers only.

^c^ Only among men diagnosed from 2012 to 2020 (*n* = 115,292).

### 3.3 Stratified Analysis

Across racial and ethnic groups, associations of insurance barriers and not having a regular provider with PSA testing with PSA screening were stronger in NHW respondents (aOR: 0.37, 95% CI: 0.34, 0.41) and Asian respondents (aOR: 0.42, 95% CI: 0.23, 0.78) and weaker for NHB (aOR: 0.63, 95% CI: 0.51, 0.78) and Hispanic (aOR: 0.50, 95% CI: 0.39, 0.64) respondents (*P*_het_ = 0.004) (Table 3). The association between unaffordable cost of visits with receipt of PSA testing did not vary statistically significantly by race (*P*_het_ = 0.067), although this association was not observed in Asians (aOR: 0.99, 95% CI: 0.57, 1.70). Associations between no regular PCP and receipt of PSA testing were weaker for NHW respondents (aOR: 0.22, 95% CI: 0.21, 0.24) compared to other racial and ethnic groups (*P*_het_ < 0.0001). After stratifying by race and ethnicity, there were no clear associations of the spatial accessibility index with receipt of PSA testing. Stronger odds ratios were reported for the association between doctor's recommendation and receipt of PSA test for Asian (aOR: 24.0, 95% CI: 14.1, 40.8) and NHW (aOR: 16.3, 95% CI: 15.2, 17.5) respondents compared to NHB (aOR: 10.8, 95% CI: 15.2, 17.5) and Hispanic (aOR: 12.0, 95% CI: 9.4, 15.4, *P*_het_ = 0.002) respondents (Table S2). Associations of access barrier did not vary by age, although inverse associations of no insurance with receipt of PSA testing were stronger among those ≥ 65 years (aOR: 0.35, 95% CI: 0.26, 0.46) compared to those < 65 years (aOR: 0.45, 95% CI: 0.41, 0.50, *P*_het_ = 0.079) (Table 4). Among those responding from 2012 to 2020, associations of doctor recommended the PSA test with screening (aOR: 16.3, 95% CI: 15.0, 17.7) were stronger in those < 65 years compared to those < 65 years (aOR: 12.7, 95% CI: 11.3, 14.4, *P*_het_ = 0.001, Table S3).

**TABLE 3:** Associations of access barriers with receipt of prostate‐specific antigen testing stratified by self‐identified race and ethnicity (2006–2020).

	NHB	Hispanic	Asian	NHW	*P*_het_
Barrier	aOR (95% CI)	aOR (95% CI)	aOR (95% CI)	aOR (95% CI)	
No insurance^a^	0.63 (0.51, 0.78)	0.50 (0.39, 0.64)	0.42 (0.23, 0.78)	0.37 (0.34, 0.41)	0.0040
Unaffordable^a^	0.81 (0.66, 0.99)	0.64 (0.50, 0.81)	0.99 (0.57, 1.70)	0.60 (0.55, 0.66)	0.067
No regular PCP^a^	0.37 (0.31, 0.46)	0.33 (0.26, 0.41)	0.37 (0.23, 0.60)	0.22 (0.21, 0.24)	< 0.0001
Doctor recommended PSA test^c^	10.8 (15.2, 17.5)	12.0 (9.4, 15.4)	24.0 (14.1, 40.8)	16.3 (15.2, 17.5)	0.002
Spatial access index^b^					0.12
Q1 (low access)	Ref	Ref	Ref	Ref	
Q2	0.92 (0.75, 1.12)	1.18 (0.91, 1.53)	0.73 (0.45, 1.19)	0.93 (0.88, 0.99)	
Q3	1.15 (0.98, 1.35)	1.08 (0.88, 1.33)	1.28 (0.83, 2.00)	0.98 (0.93, 1.03)	
Q4 (high access)	0.95 (0.80, 1.12)	0.92 (0.71, 1.19)	0.99 (0.62, 1.59)	0.87 (0.82, 0.92)	

*Note:* Unweighted samples: insurance (*n* = 184,297), unaffordable (*n* = 184,273), no regular PCP (*n* = 184,168), spatial access index (*n* = 184,643).

^a^ Logistic regression models adjusted for age, race, survey year, education, income, marital status, employment, smoking, binge drinking, reported days of poor physical health, and reported days of poor mental health, insurance, affordability, and PCP.

^b^ Models adjusted for covariates × as well as neighborhood socioeconomic status.

^c^ Only among men diagnosed from 2012 to 2020 (*n* = 115,292).

**TABLE 4:** Associations of access barriers with receipt of prostate‐specific antigen testing stratified by age (2006–2020).

	Age < 65	Age ≥ 65	*P*_het_
Barrier	aOR (95% CI)	aOR (95% CI)	
No insurance^a^	0.45 (0.41, 0.50)	0.35 (0.26, 0.46)	0.079
Unaffordable^a^	0.65 (0.59, 0.72)	0.68 (0.57, 0.82)	0.62
No regular PCP^a^	0.26 (0.24, 0.28)	0.27 (0.23, 0.32)	0.50
Doctor recommended PSA test^c^	16.3 (15.0, 17.7)	12.7 (11.3, 14.4)	0.0011
Spatial access index^b^			0.44
Q1 (low access)	Ref	Ref	
Q2	0.93 (0.87, 1.00)	0.98 (0.88, 1.09)	
Q3	1.03 (0.97, 1.00)	1.00 (0.90, 1.10)	
Q4 (high access)	0.87 (0.82, 0.93)	0.93 (0.83, 1.03)	

*Note:* Unweighted samples: insurance (*n* = 184,297), unaffordable (*n* = 184,273), no regular PCP (*n* = 184,168), spatial access index (*n* = 184,643).

^a^ Logistic regression models adjusted for age, race, survey year, education, income, census region, marital status, employment, smoking, binge drinking, reported days of poor physical health, and reported days of poor mental health, insurance, affordability, and PCP.

^b^ Models adjusted for covariates × as well as neighborhood socioeconomic status.

^c^ Only among men diagnosed from 2012 to 2020 (*n* = 115,292).

When stratifying results by education, inverse associations of costs of care (aOR: 0.34, 95% CI: 0.29, 0.40, *P*_het_ = 0.013) and perceived unaffordability (aOR: 0.56, 95% CI: 0.48, 0.65, *P*_het_ = 0.049) were strongest in college educated respondents compared to those with lower education. This pattern was also observed for the association of not having a regular PCP with screening, though did not reach statistical significance (*P*_het_ = 0.086) but not with MMSA spatial access index (Table 5). In the subset responding from 2012 to 2020, the association between doctor's recommendation and receipt of PSA test was weakest for those with less than a high school education (aOR: 11.2, 95% CI: 8.8, 14.2), but strongest for those with college or more (16.6, 95% CI: 14.9, 18.4, *P*_het_ = 0.020) (Table S4 **)**.

**TABLE 5:** Associations of access barriers with receipt of prostate‐specific antigen testing stratified by education (2006–2020).

	Less than high school	High school/some college	College or more	*P*_het_
Barrier	aOR (95% CI)	aOR (95% CI)	aOR (95% CI)	
No insurance^a^	0.44 (0.35, 0.56)	0.49 (0.43, 0.55)	0.34 (0.29, 0.40)	0.013
Unaffordable^a^	0.73 (0.59, 0.90)	0.68 (0.61, 0.77)	0.56 (0.48, 0.65)	0.049
No regular PCP^a^	0.31 (0.25, 0.39)	0.26 (0.24, 0.29)	0.24 (0.21, 0.27)	0.086
Doctor recommended PSA test^c^	11.2 (8.8, 14.2)	15.5 (14.1, 16.9)	16.6 (14.9, 18.4)	0.020
Spatial access index^b^				0.40
Q1 (low access)	Ref	Ref	Ref	
Q2	1.10 (0.88, 1.37)	0.95 (0.87, 1.04)	0.90 (0.83, 0.98)	
Q3	0.99 (0.82, 1.21)	1.03 (0.96, 1.11)	1.00 (0.93, 1.08)	
Q4 (high access)	0.91 (0.74, 1.13)	0.92 (0.86, 1.00)	0.83 (0.76, 0.90)	

*Note:* Unweighted samples: insurance (*n* = 184,297), unaffordable (*n* = 184,273), no regular PCP (*n* = 184,168), spatial access index (*n* = 184,643).

^a^ Logistic regression models adjusted for age, race, survey year, education, income, census region, marital status, employment, smoking, binge drinking, reported days of poor physical health, and reported days of poor mental health, insurance, affordability, and PCP.

^b^ Models adjusted for covariates × as well as neighborhood socioeconomic status.

^c^ Only among men diagnosed from 2012 to 2020 (*n* = 115,292).

There was no evidence for effect modification by MMSA‐level nSES for associations of insurance (*P*_het_ = 0.32) or high cost of care with receipt (*P*_het_ = 0.13) with PSA screening (Table 6). The association of not having a regular PCP with receipt of PSA testing was attenuated among those in Quintile 5 of MMSA socioeconomic status (aOR: 0.31, 95% CI: 0.26, 0.37, *P*_het_ = 0.022). There was significant effect modification for associations of the MMSA spatial access index with receipt of PSA testing (*P*_het_ < 0.0001). Higher quintile of spatial access index was associated with higher receipt of PSA testing in Quintile 5 of MMSA socioeconomic status (spatial access index Q2 aOR: 1.24, 95% CI: 1.06, 1.45; Q3 aOR: 1.32, 95% CI: 1.14, 1.53; Q4: aOR: 1.04, 95% CI: 0.90, 1.19). When restricting the population to those diagnosed between 2012 and 2020, we observed effect modification in associations of cost of care (*P*_het_ = 0.013), perceived unaffordability (*P*_het_ = 0.025), not having a regular PCP (*P*_het_ = 0.039), and spatial access index (*P*_het_ < 0.0001) with receipt of PSA testing, with inverse associations strongest in the lowest quintile of nSES (Table S4 **)**.

**TABLE 6:** Associations of access barriers with receipt of prostate‐specific antigen testing stratified by MMSA‐level nSES (2006–2020).

	nSES					*P*_het_
	Quintile 1	Quintile 2	Quintile 3	Quintile 4	Quintile 5	
Barrier	aOR (95% CI)	aOR (95% CI)	aOR (95% CI)	aOR (95% CI)	aOR (95% CI)	
No insurance^a^	0.42 (0.36, 0.48)	0.42 (0.34, 0.53)	0.40 (0.34, 0.47)	0.45 (0.37, 0.55)	0.51 (0.41, 0.62)	0.32
Unaffordable^a^	0.57 (0.50, 0.66)	0.66 (0.54, 0.81)	0.59 (0.51, 0.68)	0.65 (0.55, 0.76)	0.76 (0.63, 0.91)	0.13
No regular PCP^a^	0.27 (0.23, 0.30)	0.25 (0.21, 0.30)	0.23 (0.20, 0.27)	0.23 (0.20, 0.27)	0.31 (0.26, 0.37)	0.022
Doctor recommended PSA test^c^	14.6 (12.8, 16.6)	14.0 (12.0, 16.5)	16.0 (14.0, 18.2)	14.4 (12.5, 16.5)	16.2 (13.9, 18.9)	0.45
spatial access index^b^						< 0.0001
Q1 (low access)	Ref	Ref	Ref	Ref	Ref	
Q2	0.86 (0.77, 0.97)	0.94 (0.83, 1.07)	0.97 (0.81, 1.17)	0.77 (0.69, 0.87)	1.24 (1.06, 1.45)	
Q3	0.94 (0.86, 1.04)	0.98 (0.88, 1.09)	1.00 (0.91, 1.10)	0.84 (0.75, 0.95)	1.32 (1.14, 1.53)	
Q4 (high access)	1.10 (0.98, 1.23)	0.96 (0.82, 1.12)	0.75 (0.69, 0.82)	0.78 (0.69, 0.88)	1.04 (0.90, 1.19)	

*Note:* Unweighted samples: insurance (*n* = 184,297), unaffordable (*n* = 184,273), no regular PCP (*n* = 184,168), spatial access index (*n* = 184,643).

^a^ Logistic regression models adjusted for age, race, survey year, education, income, marital status, employment, smoking, binge drinking, reported days of poor physical health, and reported days of poor mental health, insurance, affordability, and PCP.

^b^ Models adjusted for covariates × as well as neighborhood socioeconomic status.

^c^ Only among men diagnosed from 2012 to 2020 (*n* = 115,292).

## 4 Discussion

In this study of male respondents to the BRFSS survey, we report associations of multiple access barriers (affordability, accommodation, and MMSA‐level spatial access) with receipt of PSA testing within 2 years. The strongest predictor of PSA testing was a doctor's recommendation, reflecting the importance of doctor's beliefs regarding the efficacy of screening as guidelines change over time [49]. The next strongest predictor was not having a regular PCP. Lack of insurance and cost of medical care were also associated with lower odds of PSA testing, albeit of far smaller magnitude. These findings highlight the importance of provider interactions with shared decision‐making and health system contact to influence screening behaviors in men [31, 33]. As risk‐stratified screening becomes more common, ensuring that all communities have access is important to avoid exacerbating disparities [50].

Our second goal was to examine sociodemographic variation in the importance of affordability, accommodation, and spatial accessibility barriers in relation to screening behaviors. The strength of association between access barriers and receipt of PSA testing varied by race and ethnicity, with affordability being a stronger predictor of receipt of PSA testing among NHW and Asian participants than NHB and Hispanic participants. Higher prevalence of these barriers reported by Hispanic and Black participants compared to Asian and White participants, consistent with reports of overall higher prevalence of screening among NHB and NHW populations compared to Asian and Hispanic groups [30, 51]. Research conducted in Black men at risk of CaP has revealed how community and social networks, access to information about CaP risk, and being treated with respect by healthcare providers serve as important facilitators for engaging in screening [52, 53, 54]. A weaker association between doctor's recommendation and PSA test receipt in Black compared to White men was also observed by Sammon and colleagues [31]. Ogunsanya et al. analyzed predictors of CaP screening using a different health services analytical framework, but arrived at similar conclusions regarding the importance of physician's recommendation, and discussion of benefits and harms of screening on receipt of PSA screening [33].

Although higher educational attainment is a strong predictor of use of preventive services [52, 55], education was a less important modifier than race of associations between barriers and receipt of PSA testing. Affordability and insurance were stronger predictors of PSA testing in more educated respondents, with presence of these barriers associated with lower receipt of PSA testing. Federal policies to eliminate affordability barriers through Medicaid expansion have found smaller impacts for cancer screening relative to other outcomes, but may have greater impacts in more socioeconomically vulnerable groups [56, 57, 58]. We found that MMSA‐level socioeconomic status modified the associations between having a regular PCP and spatial access index with receipt of PSA testing. The association between spatial access index and PSA testing was only observed in those in the highest quintile of MMSA socioeconomic status, and interestingly, higher spatial access index was associated with lower screening in those with MMSA quintiles 3 and 4. This non‐linear relationship may have been observed due to the imprecise assessment of neighborhood‐level socioeconomic status. Future studies that can obtain more precise estimates of spatial accessibility using travel burden models may offer more conclusive results of the importance of travel burden.

## 5 Limitations

Because this is a panel study, all reported associations between healthcare access barriers and screening behaviors are cross‐sectional. Changes in BRFSS methodology preclude assessment of time‐varying changes in screening, but we controlled for important health behaviors and sociodemographic factors that may predict screening during each survey year. The BRFSS does not release small area geographic identifiers, and so our geospatial measures were linked at the MMSA‐level, which may not accurately reflect individuals' neighborhood environments or travel burden and may have introduced measurement error [59]. However, by sampling participants across the United States, we were able to capture geographic and historical variability in health systems and socioeconomic contexts. Race and ethnicity are imputed for many respondents in the BRFSS, which may contribute to misclassification of this modifier in our analysis [60]. The sampling design of the BRFSS, which relies on phone‐based surveys, means that the experiences of the sampled population may not be truly generalizable to the US population. Future studies that oversample racialized minorities, including Indigenous Americans and Pacific Islanders, are needed to precisely estimate the prevalence of barriers and their potential influences on receipt of PSA screening. Recall bias may be correlated with healthcare use and may have led to misclassification bias in responses to questions about timing and receipt of PSA screening.

## 6 Conclusion

In this nation‐wide panel study, we studied multiple access barriers that influence receipt of PSA testing. Individual‐level self‐identified race and ethnicity and education‐level modified the associations of multiple barriers to access and receipt of PSA testing, independent of behavioral risk factors, age, and survey year. These findings imply that efforts to expand appropriate screening for men at risk of CaP are driven in large part through engagement with PCPs. Efforts to increase engagement with PCPs among socioeconomically disadvantaged groups, possibly through interventions tailored to their particular cultural and material needs, may improve access to PSA screening.

## Author Contributions

**Hari S. Iyer:** conceptualization (lead), formal analysis (lead), funding acquisition (equal), methodology (lead), writing – original draft (lead), writing – review and editing (equal). **Kevin H. Kensler:** formal analysis (supporting), writing – review and editing (equal). **Charlotte Roscoe:** funding acquisition (supporting), writing – review and editing (equal). **Chidinma Opara:** data curation (supporting), writing – review and editing (equal). **Mingchao He:** formal analysis (supporting), writing – review and editing (equal). **Evan Kovac:** writing – review and editing (equal). **Isla P. Garraway:** writing – review and editing (equal). **Quoc Dien‐Trinh:** formal analysis (supporting), writing – review and editing (equal). **Timothy R. Rebbeck:** conceptualization (supporting), formal analysis (supporting), funding acquisition (equal), methodology (supporting), writing – review and editing (equal).

## Conflicts of Interest

The authors declare no conflicts of interest.

## Supporting information


Data S1.

## Data Availability

Data used in this study are publicly available through the Centers for Disease Control and Prevention Behavioral Risk Factor Surveillance System website, which can be accessed here: https://www.cdc.gov/brfss/index.html. Analysis code is available on request to the corresponding author.
