# Supplementary material for: Multidimensional Healthcare Access Barriers to Prostate‐Specific Antigen Testing: A Nation‐Wide Panel Study in the United States From 2006 to 2020
Source: Cancer Med. 2024 Nov 6;13(21):e70358. doi: 10.1002/cam4.70358 (PMC11538963; doi:10.1002/cam4.70358)
Supplement: Supplementary file 1 — Data S1. [file CAM4-13-e70358-s001.docx]

**Multidimensional healthcare access barriers to prostate specific antigen testing: A nation-wide panel study in the United States from 2006-2020**

Authors: Hari S. Iyer, Kevin H. Kensler, Charlotte Roscoe, Chidinma Opara, Mingchao He, Evan Kovac, Isla P. Garraway, Quoc Dien-Trinh, Timothy R. Rebbeck

**Table of Contents**

**Table S1. Associations of individual-level barriers to accessing care and receipt of PSA test within 2 years in single and joint exposure models (2012-2020)**

**Table S2. Associations of access barriers with receipt of prostate-specific antigen testing stratified by self-identified race and ethnicity (2012-2020)**

**Table S3. Associations of access barriers with receipt of prostate-specific antigen testing stratified by age (2012-2020)**

**Table S4. Associations of access barriers with receipt of prostate-specific antigen testing stratified by education (2012-2020)**

**Table S5. Associations of access barriers with receipt of prostate-specific antigen testing stratified by MMSA-level nSES (2012-2020)**

**Table S1. Associations of individual-level barriers to accessing care and receipt of PSA test within 2 years in single and joint exposure models (2012-2020)**

|  | **Single** | | **Multiple** | |
| --- | --- | --- | --- | --- |
|  | **Unadjusted** | **Adjusted*** | **Unadjusted**** | **Adjusted*** |
| **Barrier** | **OR (95% CI)** | **aOR (95%C CI)** | **aOR (95% CI)** | **aOR (95%C CI)** |
| No insurance | 0.30 (0.27, 0.34) | 0.47 (0.41, 0.53) | 0.60 (0.52, 0.71) | 0.72 (0.61, 0.84) |
| Unaffordable | 0.47 (0.43, 0.52) | 0.70 (0.63, 0.78) | 0.67 (0.60, 0.76) | 0.83 (0.73, 0.94) |
| No regular PCP | 0.22 (0.20, 0.24) | 0.28 (0.26, 0.31) | 0.35 (0.31, 0.39) | 0.38 (0.34, 0.42) |
| Doctor recommended PSA test | 17.82 (16.67, 19.06) | 15.12 (14.12, 16.2) | 16.44 (15.36, 17.6) | 14.55 (13.57, 15.6) |
| **Spatial Access Index** |  |  |  |  |
| Q1 (Low access) | Ref | Ref | Ref | Ref |
| Q2 | 0.91 (0.85, 0.97 | 0.93 (0.87, 0.99 | 0.92 (0.84, 1.00) | 0.95 (0.86, 1.04) |
| Q3 | 0.93 (0.87, 0.98 | 0.94 (0.87, 1.01 | 0.92 (0.85, 1.00) | 1.03 (0.95, 1.12) |
| Q4 (High access) | 0.91 (0.85, 0.97 | 1.05 (0.98, 1.12 | 0.80 (0.73, 0.87) | 0.83 (0.76, 0.91) |

*Logistic regression models adjusted for age, race, survey year, education, income, marital status, employment, smoking, binge drinking, reported days of poor physical health, and reported days of poor mental health, census divisions

**Other barriers only

**Table S2. Associations of access barriers with receipt of prostate-specific antigen testing stratified by self-identified race and ethnicity (2012-2020)**

|  | **NHB** | **Hispanic** | **Asian** | **NHW** | ***P*_het_** |
| --- | --- | --- | --- | --- | --- |
| **Barrier** | **aOR (95% CI)** | **aOR (95% CI)** | **aOR (95% CI)** | **aOR (95% CI)** |  |
| No insurance* | 0.55 (0.42, 0.72) | 0.62 (0.46, 0.85) | 0.56 (0.24, 1.28) | 0.37 (0.33, 0.42) | <.0001 |
| Unaffordable* | 0.88 (0.69, 1.12) | 0.65 (0.49, 0.88) | 1.27 (0.67, 2.42) | 0.62 (0.56, 0.69) | <.0001 |
| No regular PCP* | 0.37 (0.29, 0.48) | 0.36 (0.27, 0.47) | 0.47 (0.27, 0.82) | 0.24 (0.21, 0.26) | <.0001 |
| Doctor recommended PSA test* | 10.8 (15.2, 17.5) | 12.0 (9.4, 15.4) | 24.0 (14.1, 40.8) | 16.3 (15.2, 17.5) | 0.002 |
| Spatial Access Index** |  |  |  |  | 0.55 |
| Q1 (Low access) | Ref | Ref | Ref | Ref |  |
| Q2 | 0.95 (0.75, 1.22) | 1.16 (0.82, 1.65) | 0.71 (0.39, 1.29) | 0.92 (0.85, 0.99) |  |
| Q3 | 1.11 (0.91, 1.35) | 1.31 (1.02, 1.67) | 1.19 (0.69, 2.04) | 0.99 (0.93, 1.06) |  |
| Q4 (High access) | 0.92 (0.74, 1.14) | 0.99 (0.71, 1.37) | 0.99 (0.56, 1.77) | 0.86 (0.81, 0.93) |  |

Unweighted samples: Insurance (n=115,052), Unaffordable (n=115,048), No regular PCP (n=114,969), doctor recommendation (N=115,292), Spatial Access Index (N=115,292).

*Logistic regression models adjusted for age, race, survey year, education, income, marital status, employment, smoking, binge drinking, reported days of poor physical health, and reported days of poor mental health, insurance, affordability, PCP, and doctors recommend for PSA test

**Models adjusted for covariates* as well as neighborhood socioeconomic status

**Table S3. Associations of access barriers with receipt of prostate-specific antigen testing stratified by age (2012-2020)**

|  | **Age <65** | **Age 65+** | ***P*_het_** |
| --- | --- | --- | --- |
| **Barrier** | **aOR (95% CI)** | **aOR (95% CI)** |  |
| No insurance* | 0.47 (0.42, 0.54) | 0.43 (0.31, 0.60) | 0.58 |
| Unaffordable* | 0.70 (0.62, 0.79) | 0.68 (0.55, 0.84) | 0.78 |
| No regular PCP* | 0.27 (0.25, 0.31) | 0.30 (0.25, 0.35) | 0.47 |
| Doctor recommended PSA test* | 16.3 (15.0, 17.7) | 12.7 (11.3, 14.4) | 0.0011 |
| Spatial Access Index** |  |  | 0.43 |
| Q1 (Low access) | Ref | Ref |  |
| Q2 | 0.91 (0.83, 1.00) | 0.99 (0.88, 1.13) |  |
| Q3 | 1.06 (0.98, 1.14) | 1.03 (0.92, 1.15) |  |
| Q4 (High access) | 0.87 (0.80, 0.95) | 0.93 (0.82, 1.06) |  |

Unweighted samples: Insurance (n=115,052), Unaffordable (n=115,048), No regular PCP (n=114,969), doctor recommendation (N=115,292), Spatial Access Index (N=115,292).

*Logistic regression models adjusted for age, race, survey year, education, income, census region, marital status, employment, smoking, binge drinking, reported days of poor physical health, and reported days of poor mental health, insurance, affordability, PCP

**Models adjusted for covariates* as well as neighborhood socioeconomic status

**Table S4. Associations of access barriers with receipt of prostate-specific antigen testing stratified by education (2012-2020)**

|  | **Less than high school** | **High school/some college** | **College+** | ***P*_het_** |
| --- | --- | --- | --- | --- |
| **Barrier** | **aOR (95% CI)** | **aOR (95% CI)** | **aOR (95% CI)** |  |
| No insurance* | 0.50 (0.37, 0.67) | 0.49 (0.41, 0.57) | 0.38 (0.31, 0.47) | 0.32 |
| Unaffordable* | 0.71 (0.55, 0.91) | 0.72 (0.62, 0.83) | 0.66 (0.56, 0.79) | 0.81 |
| No regular PCP* | 0.34 (0.26, 0.44) | 0.26 (0.23, 0.30) | 0.29 (0.25, 0.33) | 0.17 |
| Doctor recommended PSA test* | 11.2 (8.8, 14.2) | 15.5 (14.1, 16.9) | 16.6 (14.9, 18.4) | 0.020 |
| Spatial Access Index** |  |  |  | 0.58 |
| Q1 (Low access) | Ref | Ref | Ref |  |
| Q2 | 1.00 (0.75, 1.33) | 0.95 (0.85, 1.06) | 0.90 (0.82, 1.00) |  |
| Q3 | 1.15 (0.90, 1.45) | 1.05 (0.96, 1.15) | 1.01 (0.92, 1.11) |  |
| Q4 (High access) | 0.89 (0.67, 1.17) | 0.93 (0.84, 1.02) | 0.83 (0.75, 0.92) |  |

Unweighted samples: Insurance (n=115,052), Unaffordable (n=115,048), No regular PCP (n=114,969), doctor recommendation (N=115,292), Spatial Access Index (N=115,292).

*Logistic regression models adjusted for age, race, survey year, education, income, census region, marital status, employment, smoking, binge drinking, reported days of poor physical health, and reported days of poor mental health, insurance, affordability, PCP, and doctors recommend for PSA test

**Models adjusted for covariates* as well as neighborhood socioeconomic status

**Table S5. Associations of access barriers with receipt of prostate-specific antigen testing stratified by MMSA-level nSES (2012-2020)**

|  | **nSES** | | | | |  |
| --- | --- | --- | --- | --- | --- | --- |
|  | **Q1** | **Q2** | **Q3** | **Q4** | **Q5** | ***P*_het_** |
| **Barrier** | **aOR (95% CI)** | **aOR (95% CI)** | **aOR (95% CI)** | **aOR (95% CI)** | **aOR (95% CI)** |  |
| No insurance* | 0.42 (0.34, 0.51) | 0.39 (0.28, 0.53) | 0.41 (0.34, 0.51) | 0.44 (0.33, 0.57) | 0.66 (0.51, 0.86) | 0.013 |
| Unaffordable* | 0.58 (0.48, 0.69) | 0.69 (0.54, 0.89) | 0.61 (0.51, 0.73) | 0.64 (0.53, 0.77) | 0.89 (0.71, 1.12) | 0.025 |
| No regular PCP* | 0.27 (0.23, 0.32) | 0.24 (0.20, 0.30) | 0.26 (0.22, 0.31) | 0.26 (0.22, 0.32) | 0.35 (0.28, 0.43) | 0.039 |
| Doctor recommended PSA test* | 14.6 (12.8, 16.6) | 14.0 (12.0, 16.5) | 16.0 (14.0, 18.2) | 14.4 (12.5, 16.5) | 16.2 (13.9, 18.9) | 0.45 |
| Spatial Access Index** |  |  |  |  |  | <.0001 |
| Q1 (Low access) | Ref | Ref | Ref | Ref | Ref |  |
| Q2 | 0.82 (0.71, 0.95) | 1.00 (0.86, 1.17) | 0.98 (0.72, 1.35) | 0.77 (0.66, 0.90) | 1.21 (1.00, 1.46) |  |
| Q3 | 0.91 (0.81, 1.04) | 1.06 (0.93, 1.22) | 0.93 (0.83, 1.05) | 0.88 (0.75, 1.02) | 1.44 (1.20, 1.72) |  |
| Q4 (High access) | 1.07 (0.93, 1.23) | 0.85 (0.68, 1.06) | 0.72 (0.65, 0.81) | 0.72 (0.61, 0.85) | 1.12 (0.95, 1.33) |  |

Unweighted samples: Insurance (n=115,052), Unaffordable (n=115,048), No regular PCP (n=114,969), doctor recommendation (N=115,292), Spatial Access Index (N=115,292).

*Logistic regression models adjusted for age, race, survey year, education, income, marital status, employment, smoking, binge drinking, reported days of poor physical health, and reported days of poor mental health, insurance, affordability, PCP, and doctors recommend for PSA test

**Models adjusted for covariates* as well as neighborhood socioeconomic status
